# SPATA2-Mediated Binding of CYLD to HOIP Enables CYLD Recruitment to Signaling Complexes

**DOI:** 10.1016/j.celrep.2016.07.086

**Published:** 2016-08-18

**Authors:** Sebastian Kupka, Diego De Miguel, Peter Draber, Luigi Martino, Silvia Surinova, Katrin Rittinger, Henning Walczak

**Affiliations:** 1 Centre for Cell Death, Cancer, and Inflammation (CCCI), UCL Cancer Institute, University College London, 72 Huntley Street, London WC1E 6DD, UK; 2 The Francis Crick Institute, 1 Midland Road, London NW1 1BF, UK

## Abstract

Recruitment of the deubiquitinase CYLD to signaling complexes is mediated by its interaction with HOIP, the catalytically active component of the linear ubiquitin chain assembly complex (LUBAC). Here, we identify SPATA2 as a constitutive direct binding partner of HOIP that bridges the interaction between CYLD and HOIP. SPATA2 recruitment to TNFR1- and NOD2-signaling complexes is dependent on HOIP, and loss of SPATA2 abolishes CYLD recruitment. Deficiency in SPATA2 exerts limited effects on gene activation pathways but diminishes necroptosis induced by tumor necrosis factor (TNF), resembling loss of CYLD. In summary, we describe SPATA2 as a previously unrecognized factor in LUBAC-dependent signaling pathways that serves as an adaptor between HOIP and CYLD, thereby enabling recruitment of CYLD to signaling complexes.

## Highlights

• SPATA2 bridges the interaction between HOIP and CYLD
• CYLD recruitment to the TNFR1-signaling complex requires SPATA2
• Loss of SPATA2 phenocopies absence of CYLD in TNFR1 signaling

## Graphical Abstract

**Figure:**
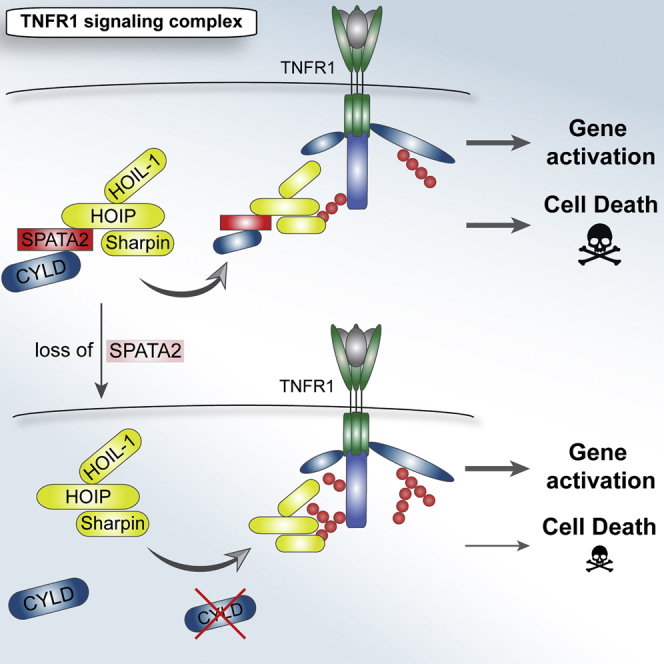

## Teaser

Kupka et al. show that the previously demonstrated interaction of CYLD with HOIP, which is required for recruitment of CYLD into signaling complexes, is indirect and mediated by SPATA2. Loss of SPATA2 abrogates recruitment of CYLD to signaling complexes and consequently mimics CYLD deficiency with regards to gene activation and necroptosis induced by TNF.

## Introduction

Over the past few years, the balancing roles of E3 ubiquitin ligases (E3s) and deubiquitinases (DUBs) in creating and degrading ubiquitin chains, respectively, have emerged as crucial at regulating innate and adaptive immune responses (Fiil and Gyrd-Hansen, 2014, Zinngrebe et al., 2014). There are eight different kinds of ubiquitin chains that accomplish different physiological outcomes (Yau and Rape, 2016). For example, lysine 48 (K48)-linked chains target proteins for degradation by the proteasome, whereas K63- and methionine 1 (M1)-linked chains (the latter also referred to as linear ubiquitin chains) are involved in the regulation of gene activation pathways and cell death (Chen and Sun, 2009, Iwai et al., 2014). The differently linked types of ubiquitin chains are generated by specific E3s and are degraded by specialized DUBs. Hence, precise timing of the respective activities of these enzymes is paramount for fine regulation of the signaling output generated by ubiquitin-involving signaling complexes (SCs) (Chen and Sun, 2009, Kupka et al., 2016, Zinngrebe et al., 2014).

Tumor necrosis factor (TNF) binding to TNF receptor 1 (TNFR1) triggers formation of the TNFR1 signaling complex (TNFR1-SC) (Walczak et al., 2012). Signals initiated from this complex result in two very different outcomes: (1) induction of gene activation via NF-κB and mitogen-activated protein (MAP) kinases and (2) induction of cell death, which can either be apoptotic or necroptotic. Linear ubiquitination, mediated by the linear ubiquitin chain assembly complex (LUBAC), is crucial in deciding the fate of cells upon TNF stimulation. In the absence of LUBAC, the lack of linear ubiquitin chains in the TNFR1-SC results in defective recruitment of various components and complex destabilization (Haas et al., 2009). This shifts the signaling toward enhanced formation of a secondary SC, which induces cell death (Peltzer et al., 2014), also referred to as complex II of TNFR1 signaling (Newton and Manning, 2016). In addition, linear and other ubiquitin linkages are removed by the DUB CYLD, a process that is crucial to enable the formation of complex II, as CYLD- deficient cells are resistant to TNF-induced cell death (Draber et al., 2015, Moquin et al., 2013).

LUBAC targets within the TNFR1-SC include RIP1, NEMO, TNFR1, and TRADD (Draber et al., 2015, Gerlach et al., 2011, Tokunaga et al., 2011). Furthermore, LUBAC regulates signaling through various other receptors, including CD40, NOD2, and IL-1R (Damgaard et al., 2012, Emmerich et al., 2013, Gerlach et al., 2011). LUBAC is composed of three subunits: SHARPIN, HOIL-1, and the catalytic component HOIL-1 interacting protein (HOIP) (Draber et al., 2015, Haas et al., 2009, Ikeda et al., 2011, Kirisako et al., 2006, Tokunaga et al., 2011). Additionally, LUBAC is associated with two DUBs: CYLD and OTULIN (Draber et al., 2015, Elliott et al., 2014, Takiuchi et al., 2014). Interaction of OTULIN and CYLD with HOIP is mutually exclusive (Draber et al., 2015). Although CYLD is co-recruited into signaling complexes via HOIP, OTULIN is not (Draber et al., 2015). The mechanistic explanation for this observation remains elusive, yet together, these findings point toward specific and distinct functions for OTULIN versus CYLD in regulating LUBAC.

Intriguingly, although the interaction of OTULIN with HOIP has been shown to be direct and was structurally characterized (Elliott et al., 2014, Schaeffer et al., 2014), we were not able to detect direct binding of CYLD to HOIP. This suggested the existence of (an) additional factor(s) mediating this interaction. The search for such a factor resulted in the discovery of SPATA2 as a previously unrecognized component of the TNFR1-SC and NOD2-SC, which bridges the interaction between CYLD and HOIP by directly interacting, via distinct domains, with both proteins.

## Results

### SPATA2 Is a Component of the TNFR1-SC

**Figure 1:**
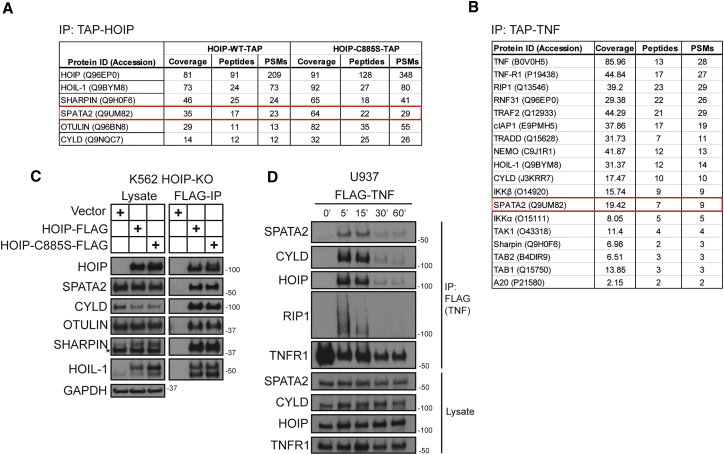
SPATA2 Constitutively Interacts with LUBAC and Forms Part of the Native TNFR1-SC (A) HOIP-deficient K562 cells were reconstituted with TAP-tagged wild-type (WT) HOIP or enzymatically inactive HOIP-C885S. HOIP-containing complexes were purified and analyzed by mass spectrometry. (B) TNFR1-SC was purified from A549 cells using TAP-TNF (500 ng/ml). Protein complexes were analyzed by mass spectrometry. (C) Samples were prepared as in (A) and subjected to western blot analysis. (D) U937 cells were stimulated with FLAG-TNF (500 ng/ml) for the indicated time. TNFR1-SC was purified using anti-FLAG beads and analyzed by western blotting.

To address whether there may be factors, in addition to CYLD, that are constitutively associated with LUBAC and recruited to the TNFR1-SC, we performed two different mass spectrometry (MS) analyses using a modified tandem affinity purification (TAP) approach. In the first one, we employed TAP-tagged TNF (TAP-TNF) to identify components of the TNFR1-SC and in the second one we expressed TAP-tagged HOIP (HOIP-TAP). CYLD, and the other two LUBAC components, HOIL-1 and SHARPIN, served as positive controls in this analysis; indeed, all three factors were identified in both proteomic approaches as high-scoring interactors. SPATA2 was an additional protein that was identified by both proteomic approaches (Figures 1A and 1B; Tables S1 and S2). Thus, SPATA2 was a candidate for a constitutive interaction partner of LUBAC and for a previously unrecognized component of the TNFR1-SC.

We validated the constitutive interaction of SPATA2 with HOIP by western blotting (Figure 1C). When examining whether SPATA2 formed part of the native TNFR1-SC, we found that it was recruited to this complex. In line with the constitutive interaction between SPATA2 and LUBAC, this occurred with kinetics similar to those of HOIP and CYLD (Figure 1D). Hence, SPATA2 is a previously unrecognized component of the native TNFR1-SC and a constitutive interaction partner of LUBAC.

### SPATA2 Recruitment to the TNFR1- and NOD2-SC Requires HOIP

**Figure 2:**
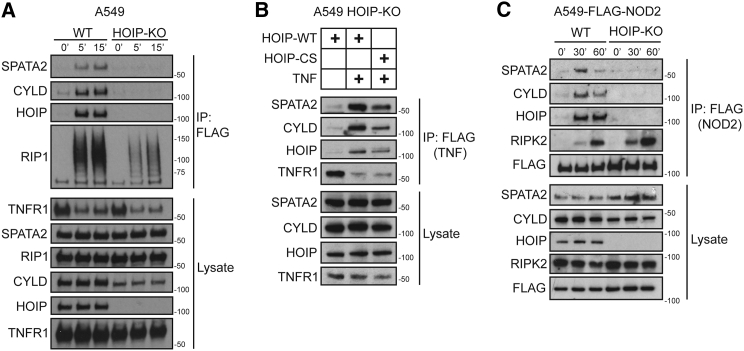
SPATA2 Is Recruited to the TNFR1-SC and NOD2-SC via HOIP (A) WT or HOIP-deficient A549 cells were stimulated with FLAG-TNF (500 ng/ml) for the indicated times. TNFR1-SC was purified and analyzed by western blotting. (B) HOIP-deficient A549 cells were reconstituted with either HOIP-WT or HOIP-CS, and TNFR1-SC was purified as in (A). (C) Wild-type (WT) or HOIP-deficient A549 cells were stably transfected with NOD2-TAP, stimulated with L18-MDP (200 ng/ml) for the indicated times, and analyzed by western blotting.

We next tested whether SPATA2 was recruited to the TNFR1-SC due to its constitutive interaction with HOIP, in a manner similar to CYLD (Draber et al., 2015). This analysis revealed that SPATA2 was absent from the TNFR1-SC in HOIP-deficient A549 cells (Figure 2A), indicating that recruitment of SPATA2 is dependent on HOIP.

Despite the constitutive interaction between SPATA2 and LUBAC, it was possible that linear ubiquitination could be required for its recruitment to, or retention in, the TNFR1-SC. This was, however, not the case, as SPATA2 recruitment was unaltered in HOIP-deficient A549 cells reconstituted with an inactive form of HOIP (HOIP-C885S) (Figure 2B). Hence, SPATA2 recruitment to the TNFR1-SC requires LUBAC, but not its catalytic activity.

We previously showed that, in addition to the TNFR1-SC, LUBAC also forms part of various other signaling complexes, including the NOD2-SC (Damgaard et al., 2012, Draber et al., 2015, Gerlach et al., 2011, Haas et al., 2009). We therefore tested whether SPATA2 is also recruited to the NOD2-SC and, if so, whether its recruitment requires HOIP. When stimulating A549 cells expressing FLAG-tagged NOD2 with L18-MDP and purifying the resulting signaling complex, we found that SPATA2 was readily recruited alongside HOIP and CYLD (Figure 2C). Importantly, SPATA2, like CYLD, was not recruited to the NOD2-SC in cells devoid of HOIP (Figure 2C). Hence, SPATA2 forms part of the NOD2-SC, and its recruitment is a result of its constitutive interaction with LUBAC via HOIP.

### SPATA2 Is Required for Recruitment of CYLD to the TNFR1-SC

**Figure 3:**
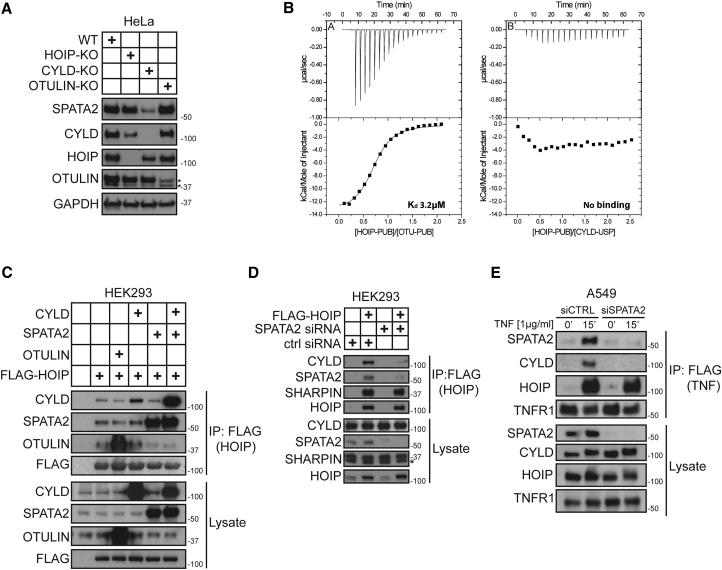
Binding of CYLD to HOIP and Its Recruitment to the TNFR1-SC Requires SPATA2 (A) HOIP, CYLD, or OTULIN were knocked out via clustered regularly interspaced short palindromic repeats (CRISPR)-Cas9 and levels of the indicated proteins compared to HeLa WT by western blot analysis. (B) ITC characterization of the interactions between (A) the HOIP-PUB domain (597 μM) and a peptide derived from the PIM motif (58 μM) of OTULIN and (B) the HOIP-PUB domain (398 μM) and the CYLD-USP domain (40 μM). For each titration, the raw data and normalized integrated heats are reported. (C) HEK293 cells were transfected with different combinations of CYLD, SPATA2, and OTULIN together with FLAG-HOIP. Protein complexes were subsequently purified using anti-FLAG beads and analyzed by western blotting. (D) SPATA2 expression was suppressed in FLAG-HOIP transfected HEK293 cells using siRNA. FLAG-HOIP was subsequently immunoprecipitated and tested for associated proteins by western blot analysis. (E) A549 cells were transfected with control or SPATA2 siRNA. After 72 hr, cells were subjected to TNFR1-SC purification and analyzed by western blotting.

Comparison of protein levels in different knockout cells showed that SPATA2 levels were drastically reduced in HeLa cells deficient in CYLD but not in cells deficient in OTULIN or HOIP (Figure 3A). This suggested that CYLD and SPATA2 might be functionally connected and possibly interact with each other.

Previous data showed that the USP domain of CYLD is associated with the PUB domain of HOIP (Takiuchi et al., 2014). However, we could not detect a direct interaction between these domains by isothermal titration calorimetry (ITC) (Figure 3B). Additionally, when we overexpressed CYLD in HOIP-TAP-expressing cells, we found that overexpression only marginally increased the amount of CYLD bound to HOIP as compared to endogenous levels of CYLD (Figure 3C). This is in stark contrast to the interaction of HOIP with OTULIN, which was substantially increased by OTULIN overexpression (Figure 3C). This suggested that the interaction between HOIP and CYLD is mediated by an additional factor, and we were therefore prompted to test whether SPATA2 may function as an adaptor. If this were the case, the interaction of HOIP and CYLD should increase as a function of co-overexpression of SPATA2. Strikingly, the association of CYLD with HOIP drastically increased when SPATA2 was also overexpressed (Figure 3C). Accordingly, when we reduced SPATA2 levels by RNAi and immunoprecipitated overexpressed FLAG-tagged HOIP, the amount of CYLD associated with HOIP was drastically decreased (Figure 3D).

We next tested whether SPATA2 is required for recruitment of CYLD to the TNFR1-SC. Analysis of the TNFR1-SC showed that, compared to control cells, CYLD was virtually absent from the TNFR1-SC that formed in cells in which SPATA2 expression was suppressed by RNAi (Figure 3E). Together, this demonstrates that SPATA2 serves as an adaptor between CYLD and HOIP and that SPATA2 is required and sufficient for recruitment of CYLD, via HOIP, to the TNFR1-SC.

### The N Terminus of SPATA2 Interacts with the USP Domain of CYLD, whereas Its C Terminus Binds to the PUB Domain of HOIP

**Figure 4:**
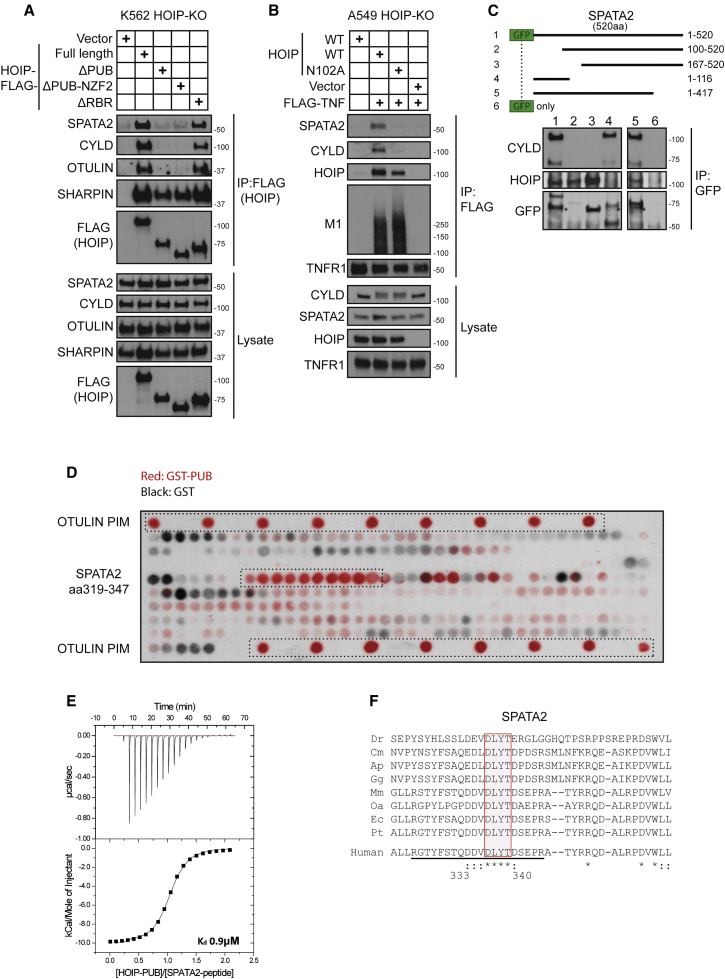
A PIM in SPATA2 Interacts with the PUB Domain of HOIP, whereas Its N Terminus Binds to CYLD (A) HOIP-deficient K562 cells reconstituted with vector control, FLAG-HOIP-WT, or different truncated versions of FLAG-HOIP were lysed and subjected to immunoprecipitation (IP) of HOIP. Samples were subsequently analyzed by western blotting. (B) HOIP-deficient A549 cells were reconstituted with HOIP-WT or HOIP-N102A. Cells were stimulated with FLAG-TNF and the resulting signaling complexes subsequently purified using anti-FLAG IP. Samples were analyzed by western blotting. (C) Truncated forms of GFP-SPATA2 were expressed in HEK293 cells. Following anti-GFP IP, SPATA association with HOIP and CYLD was evaluated by western blot analysis. (D) Peptide arrays representing amino acids 200–520 of SPATA2 as a series of 20-mer overlapping peptides, shifted by one, blotted on nitrocellulose membranes. The OTULIN PIM peptide served as a positive control. One membrane was incubated with recombinant GST-only (GST) the other one with GST-tagged PUB-domain of HOIP (GST-PUB). Bound GST-containing protein was subsequently detected by HRP immunofluorescence, and the signal of both membranes was overlaid. (E) ITC characterization of the interaction between HOIP-PUB domain (500 μM) and a peptide (50 μM) derived from the C-terminal region of SPATA2 (RGTYFSTQDDVDLYTDSEPR). The raw data and normalized integrated heats are reported. (F) Sequence alignment of the SPATA2 PIM region in different species. Underlined sequence indicates the peptide used in (E). Dr, *Danio rerio*; Cm, *Chelonia mydas*; Ap, *Anas platyrhynchos*; Gg, *Gallus gallus*; Mm, *Mus musculus*; Oa, *Ovis aries*; Ec, *Equus caballus*; Pt, *Pan troglodytes*; Hs, *Homo sapiens*.

We and others previously showed that the (PUB)-domain of HOIP is essential for recruitment of CYLD (Draber et al., 2015, Elliott et al., 2014). To test whether SPATA2 interacts with this domain, we reconstituted HOIP-deficient K562 cells with FLAG-tagged truncated versions of HOIP and immunoprecipitated the associated complex. This revealed that only constructs encompassing the N-terminal PUB domain bound SPATA2 (Figure 4A). Additionally, we used a HOIP-PUB domain point mutant (N102A), which abolishes the interaction of both OTULIN and CYLD with HOIP (Draber et al., 2015, Elliott et al., 2014). Importantly, this mutant was also unable to restore SPATA2 recruitment to the TNFR1-SC (Figure 4B). Together, these data indicate that the PUB domain of HOIP is essential for the interaction with SPATA2 and, consequently, for recruitment of CYLD to the TNFR1-SC.

To characterize the interaction of SPATA2 with CYLD and HOIP further, we expressed various deletion mutants of SPATA2 with a GFP tag. Immunoprecipitation of these truncated forms of SPATA2 revealed that the interaction with CYLD is mediated by the N terminus of SPATA2 as a fragment comprising amino acids 1–116 was sufficient to bind to CYLD (Figure 4C). In contrast, only SPATA2 fragments comprising amino acids 167–417 bound to HOIP (Figure 4C).

### SPATA2 Contains a PIM that Mediates the Interaction with the PUB Domain of HOIP

Because the N102A mutation in the PUB domain of HOIP abolishes the interaction with OTULIN and SPATA2, we assumed that, similar to OTULIN, SPATA2 might also contain a PUB-domain interacting motif (PIM). To precisely map the interacting peptide, we designed a peptide array, spotted on a nitrocellulose membrane, composed of 20-mer peptides that overlap by one amino acid and span amino acids 201–520 of SPATA2. Incubation with glutathione S-transferase (GST) or GST-tagged PUB domain of HOIP identified that SPATA2 peptides corresponding to amino acids 319–347 interacted specifically with HOIP’s PUB domain (Figure 4D). The overlapping sequence between interacting peptides suggests RGTYFSTQDDVDLYTDSEPR as the PIM of SPATA2. Characterization of the interaction by ITC revealed an affinity of 0.9 μM (Figure 4E). Additionally, sequence alignment of this PIM from different species revealed a high degree of evolutionary conservation (Figure 4F).

Together, these results show that SPATA2 contains two distinct domains that are responsible for mediating the interaction with CYLD and HOIP, respectively; while the N terminus of SPATA2 binds to the USP domain of CYLD, the interaction with HOIP is mediated via a highly conserved PIM located in the central portion of SPATA2, which is recognized by the PUB domain of HOIP.

### SPATA2 or CYLD Deficiency has Limited Effects on TNF-Induced Gene Activatory Pathways

**Figure 5:**
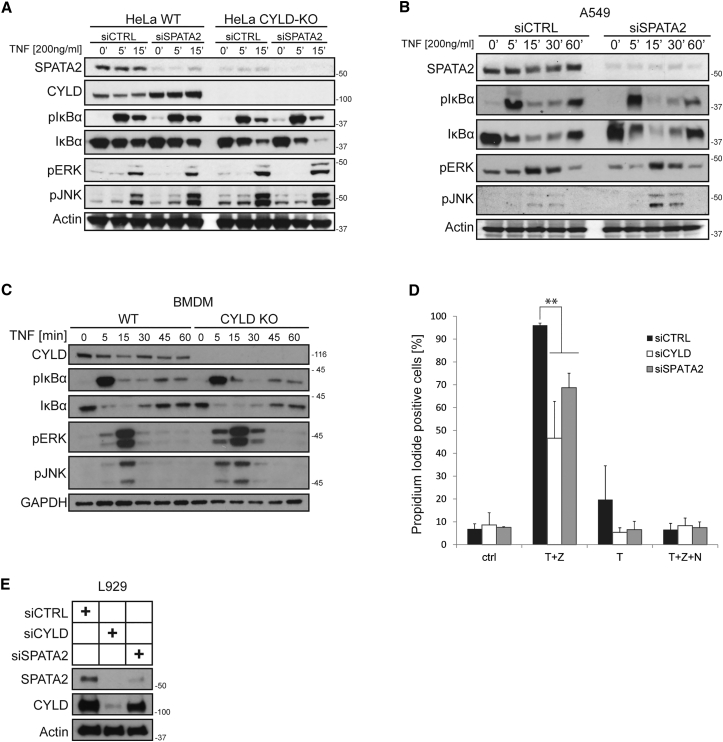
SPATA2 Deficiency Reduces TNF-Induced Necroptosis but Has Minor Effects on TNF-Mediated Gene Activation (A) SPATA2 levels were reduced by siRNA in HeLa WT and HeLa CYLD-KO cells. Cells were then stimulated with TNF for the indicated time, lysed, and subjected to western blot analysis. (B) Knockdown of SPATA2 was performed in A549 by siRNA. Cells were then stimulated with TNF for the indicated time, lysed, and subjected to western blot analysis. (C) Bone-marrow-derived macrophages were isolated from CYLD-deficient mice or wild-type littermates. Cells were then stimulated with TNF and analyzed by western blotting. (D) SPATA2 or CYLD expression in L929 was silenced by siRNA. Cells were stimulated with 20 ng/ml TNF and the indicated inhibitors. After 16 hr, cells were collected, stained with propidium iodide, and measured by fluorescence-activated cell sorting (FACS). Data are presented as mean ± SEM (n = 3); ^∗∗^p < 0.01, statistics were performed using a two-tailed, unpaired Student’s t test. (E) Representative western blot analysis of the knockdown efficiency in L929 cells used in the experiment shown in (D).

We next examined how SPATA2 knockdown affected TNF-induced gene activation. To do so, we employed wild-type and CYLD-deficient HeLa cells and depleted SPATA2 by RNAi before stimulating these cells with TNF. This analysis revealed no increase in the activation of NF-κB in SPATA2-depleted as compared to control cells, whereas activation of c-Jun N-terminal kinase (JNK) was only slightly increased (Figure 5A). However, the same was true for CYLD-deficient cells, and, importantly, no further increase was seen by concomitant SPATA2 suppression (Figure 5A). Similarly, RNAi-mediated suppression of SPATA2 in A549 cells did not increase TNF-induced activation of NF-κB, but it did increase activation of JNK (Figure 5B). Because CYLD deficiency is generally assumed to significantly affect TNF-induced NF-κB activation, we also stimulated primary bone-marrow-derived macrophages (BMDMs) with TNF and analyzed activation of JNK, extracellular signal-regulated kinase (ERK), and NF-κB. In line with our observation in cell lines, however, no difference in NF-κB activation could be detected between CYLD-proficient and CYLD-deficient BMDMs, whereas deficiency in CYLD resulted in a slight increase in JNK and ERK activation in these cells (Figure 5C). Thus, in all cell types we tested, SPATA2 or CYLD deficiency had little if any effect on TNF-induced NF-κB activation but slightly increased JNK activation.

### SPATA2 Deficiency Diminishes TNF-Induced Necroptosis

In addition to its proposed role in inhibiting gene activation induced by various ligands, CYLD has been shown to enhance TNF-mediated cell death. More specifically, necroptosis was shown to require CYLD in L929 cells (Hitomi et al., 2008, Moquin et al., 2013, O’Donnell et al., 2011). To test whether SPATA2 was also required for necroptosis, we reduced SPATA2 or CYLD expression in the murine cell line L929 using small interfering RNA (siRNA) (Figures 5D and 5E) and stimulated these cells with TNF/zVAD to induce necroptosis. This revealed that knockdown of SPATA2 protected L929 cells from TNF/zVAD-induced necroptosis to a similar extent as CYLD knockdown (Figure 5D). Thus, like CYLD, SPATA2 serves as a factor that enables TNF-induced cell death.

## Discussion

We previously showed that CYLD recruitment to the TNFR1-SC requires HOIP (Draber et al., 2015). Here, we show that SPATA2 is indispensable for CYLD recruitment to this complex by bridging CYLD and HOIP. Like OTULIN, SPATA2 directly binds to the PUB domain of HOIP, and their interactions are mutually exclusive. Because SPATA2 also requires HOIP for its recruitment to the NOD2-SC, we deem it likely that the complex consisting of LUBAC, SPATA2, and CYLD is the default complex recruited also to other receptor-associated complexes known to involve CYLD and/or LUBAC (Douanne et al., 2016, Tauriello et al., 2010). Given the growing relevance of the equilibrium between ubiquitination and deubiquitination in the regulation of signaling complexes (Harhaj and Dixit, 2012, Kupka et al., 2016, Yau and Rape, 2016) and the involvement of CYLD in a wide number of signaling platforms (Draber et al., 2015, Mathis et al., 2015, Reiley et al., 2006, Tauriello et al., 2010, Zhang et al., 2011), the discovery of SPATA2 as a previously unrecognized adaptor between CYLD and HOIP by us and others (Wagner et al., 2016) provides additional insight on the mechanisms by which this DUB controls the outcome of these diverse signaling processes.

TNF-stimulated signaling induces gene activation pathways and, under certain circumstances, programmed cell death (Sedger and McDermott, 2014, Wertz, 2014). Here, we show that in absence of SPATA2, CYLD is not recruited to the TNFR1-SC. Therefore, absence of SPATA2 should affect TNF-induced signaling in a manner similar to the absence of CYLD. Previous studies reported the importance of CYLD for induction of necroptosis (Hitomi et al., 2008, Moquin et al., 2013, O’Donnell et al., 2011). In line, siRNA-mediated knockdown of SPATA2 or CYLD protected cells from TNF-induced necroptosis to a similar extent, Likewise, suppression of SPATA2 correlated with that of CYLD regarding gene activation pathways, exerting minor effects on TNF-induced NF-κB activation but showing a slight effect on JNK activation in the different cell types tested. This is in apparent contrast to the accepted role of CYLD as a negative regulator of NF-κB activation and results presented on the role of SPATA2 in gene activation while our study was under review (Wagner et al., 2016). It should be noted, however, that most studies investigating the role of CYLD in NF-κB activation were performed using overexpression models and/or luciferase reporter assays, mostly in HEK293 cells (Brummelkamp et al., 2003, Kovalenko et al., 2003, Trompouki et al., 2003). The physiological relevance of these results is at least arguable, since they are based on artificial and isolated reporter elements. Indeed, luciferase reporter assays have already been described to be unreliable under certain circumstances, specifically in HEK293 cells (Ling et al., 2012). In fact, several studies previously reported only minimal effects of CYLD deficiency on TNF-induced gene activation in general and a slight increase of JNK activation (Moquin et al., 2013, Reiley et al., 2006, Zhang et al., 2006). Together, it appears that the role of CYLD with respect to gene activation is certainly more cell-type and pathway dependent than currently thought. Our results on SPATA2 and CYLD, in connection with the previous literature on CYLD, provide the rationale for a thorough reassessment of the effects of deficiency in CYLD, and now also of SPATA2, on gene activation induced by different stimuli and in different cell types.

Initially, SPATA2 was described as a protein that might be involved in spermatogenesis, as it is highly expressed in testis and strongly upregulated during spermatogenesis (Onisto et al., 2001). Interestingly, CYLD-deficient mice, despite having no overt phenotype, have been reported to show defects in spermatogenesis (Wright et al., 2007). In light of our result that CYLD-deficient cells have drastically reduced SPATA2 levels, it is possible that these defects are due to SPATA2 deficiency. It will be interesting to investigate the connection between CYLD and SPATA2 with regards to defects in spermatogenesis, i.e., whether in this context the function of SPATA2 is CYLD dependent.

The observation that SPATA2 plays an important role in CYLD function could be of clinical relevance, as patients with mutations in CYLD develop cylindromatosis (Biggs et al., 1995). A first analysis of the publicly available databases for autoimmune patients did not reveal any overt polymorphisms in the SPATA2 gene. However, it is possible that SPATA2 mutations could be involved in patients suffering from cylindromatosis or related diseases who do not have mutations in CYLD and for whom a molecular explanation is consequently still missing (Dubois et al., 2015, Saggar et al., 2008).

## Experimental Procedures

### Plasmids and Cloning

SPATA2 was cloned from cDNA by conventional PCR using 5′-atggggaagcccagttca-3′ as the forward and 5′-ctatctgtacacgagatgggagag-3′ as the reverse target sequence. HOIP and CYLD expression constructs were described previously (Draber et al., 2015).

### SDS-PAGE, Western Blot, and Antibodies

Proteins were separated on 4%–15% Mini-PROTEAN TGX Precast Gels with TGX running buffer. Proteins were transferred onto 0.2 μM nitrocellulose membrane (Bio-Rad, Trans-Blot Turbo Mini Nitrocellulose Transfer Packs). Proteins were detected via western blot using the following antibodies: SPATA2 (Abcam, ab56565), SPATA2 (Bethyl Laboratories, A302-494A), HOIP (Aviva System Biology, ARP43241_P050), SHARPIN (Proteintech, 14626-I-AP), HOIL-1 (Haas et al., 2009), CYLD (Santa Cruz Biotechnology, sc-74435), OTULIN (Abcam, ab151117), RIP1 (BD, 610459), TNFR1 (Santa Cruz, 8436), M1-Ub (Merck Millipore, MABS199), IκBα (Cell Signaling Technology, 9242), pIκBα (Cell Signaling, 9246S), pP65 (Cell Signaling, 7F1), pErk (Santa Cruz, SC-7383), pP38 (Cell Signaling, D3F9), pJNK (Cell Signaling, 98F2), Actin (Sigma-Aldrich, A1978), GAPDH (Abcam, ab8245), FLAG (Sigma-Aldrich, M2), and GST (Cell Signaling, 2622).

### Production of Recombinant TNF

The coding sequences of TAP-TNF, consisting of a His-tag followed by 3x FLAG tag, a PreScission cleavage site, and a 2x Strep-tag II, and the extracellular portion of TNF (aa 78–233) or His-TNF (aa 78–233) were inserted in pQE30 vector. Protein was expressed in BL21 (DE3) cells with 1 mM isopropyl β-D-1-thiogalactopyranoside (IPTG) overnight and purified by affinity chromatography on His GraviTrap TALON columns (GE Healthcare), eluted with 500 mM imidazole and dialyzed against storage buffer (50 mM Tris [pH 7.4], 100 mM NaCl, 0.02% Tween, 2 mM DTT, and 0.5 M arginine). Protein concentration was determined with a Nanodrop 2000 (Thermo Scientific) and samples were stored at −20°C.

### Transient Transfection

Cells were seeded the day before and allowed to reach ∼70% confluency on the day of transfection. For a six-well plate, 1 μg DNA was used per well. First, DNA was diluted in 100 μl Opti-MEM medium, and TurboFect transfection reagent (Thermo Scientific) or Lipofectamine 2000 (Thermo Fisher) was added in a 1:3 DNA/transfection reagent ratio. After a 30 min of incubation, the mixture was added dropwise to the cells. Cells were allowed to grow for another day before use for experiments.

### siRNA-Mediated Knockdown

Transient knockdown was performed by reverse transfection of cells with siRNA using Lipofectamine 2000 (Invitrogen) following the manufacturer’s recommendations. In summary, Lipofectamine 2000 was added to medium free of FCS and antibiotics and incubated for 20 min at room temperature. Next, siRNA was added and left at room temperature for 30 min. The reaction mix was then added dropwise to the cells, and the final volume was adjusted with antibiotic free medium. The ratio of Lipofectamine/siRNA was 3 μl/μg siRNA in all cases. All experiments were carried out at least 48 hr after the reverse transfection.

### Immunoprecipitation of Protein Complexes

For immunoprecipitation of ectopically expressed tagged proteins, cells were lysed in immunoprecipitation (IP)-lysis buffer and lysate was cleared by centrifugation. FLAG-tagged proteins were immunoprecipitated using anti-FLAG (M2) beads (Sigma-Aldrich). Where other antibodies have been used, 1 μg antibody was bound to Protein A/G beads (Santa Cruz Biotechnology) prior to the addition to the lysate. Immunoprecipitations were carried out at 4°C with gentle rotation for 4–16 hr. Beads were then washed three times with lysis buffer and reduced using LDS sample buffer.

### TNFR1-SC Purification

FLAG-tagged TNF (1 μg/ml) in medium (37°C) was added to the cells for the indicated time. Cells were left in the incubator during the course of stimulation. Subsequently, stimulation medium was aspirated and cells were washed with cold PBS. Cells were lysed in IP-lysis buffer and cellular debris was cleared by centrifugation at 13,000 rpm for 20 min. 1/100 of the amount of FLAG-TNF used for the stimulation was added to lysates from non-stimulated cells as a negative control. 10 μg M2 beads (Sigma-Aldrich) were then added to the lysate and incubated overnight at 4°C. The next day, samples were washed three times with IP buffer and then reduced in sample buffer.

### Mass Spectrometric Analysis of Immunoprecipitated Complexes

Protein complexes were processed and analyzed as in Draber et al. (2015). In brief, protein mixtures were denatured, reduced, alkylated, and digested with Lys-C and Trypsin. Desalted samples were analyzed by nLC-MS/MS on a Q Exactive Orbitrap coupled to an Easy-nLC 1000 (Thermo Scientific).

### Isolation of Bone-Marrow-Derived Macrophages

For preparation of BMDMs, 8-week-old mice were sacrificed. Hindlimbs were removed, and bones were separated from muscle tissue. Femur and tibia were opened on each site, and bone marrow was flushed out using a 25G needle and syringe. Cells were then resuspended in RPMI medium containing 10% fetal calf serum (FCS), 1% penicillin/streptomycin (Invitrogen), and 10% conditioned medium from L929 cells and passed through a cell strainer. Subsequently, cells were plated in a 12-well plate. The conditioned medium was replaced every 2 days, and cells were incubated for 7 days before the experiment.

### Cell Death Analysis

Cells were treated with 200 ng/ml TNF or in combination with 20 μM zVAD-fmk (Abcam) and/or 10 μM Nec-1 s (Biovision) for 24 hr. Supernatant was collected and remaining live cells were trypsinized. Supernatant and detached cells were combined and centrifuged at 2,500 rpm for 10 min. The pellet was then resuspended in PBS containing 5 μg/ml propidium iodide (Sigma). Cells were analyzed by FACS (BD Accuri C6 or Fortessa). Data are presented as mean ± SEM (n = 3), and statistics were performed using t tests.

### Peptide Arrays

Peptide arrays covering residues 200–520 of SPATA2 as a series of 20-mer overlapping peptides were synthesized by the Peptide Chemistry Science Technology Platform of the Francis Crick Institute. Arrays were activated with 70% ethanol for 15 min and blocked with 5% milk powder in PBS containing 0.05% Tween-20 (PBST). After blocking, the membranes were washed three times with PBST. Recombinant GST or GST-PUB-HOIP in PBST containing 2.5% BSA was added to the respective membrane. The blots were incubated for 1 hr at room temperature with gentle agitation, washed three times with PBST, and subsequently incubated with anti-GST primary antibody and detected with horseradish peroxidase (HRP)-coupled anti-rabbit antibody.

### Isothermal Titration Calorimetry

ITC experiments were performed at 293 K using an ITC-200 microcalorimeter (Malvern Instruments). The protein and peptide solutions were prepared in 25 mM HEPES (pH 7.5), 150 mM NaCl, and 0.5 mM TCEP. Titrations were performed by titrating 20 times 2 μl HOIP-PUB domain (597 μM) into the OTULIN peptide (AEHEEDMYRA) at 58 μM, 398 μM HOIP-PUB domain into the CYLD-USP domain at 40 μM, and 500 μM HOIP-PUB domain into the SPATA2 peptide (RGTYFSTQDDVDLYTDSEPR) at 50 μM. Integrated heats corrected for heats of dilution were fitted using a 1:1 binding model in the MicroCal-Origin 7.0 software package.

### Statistical Procedures

Where indicated, data from at least three biological replicates are presented as mean ± SEM. Statistical significance was calculated by performing a two-tailed, unpaired Student’s t test, with ^∗∗^ indicating p ≤ 0.01.

## Author Contributions

H.W. conceived the project. S.K., D.D.M., and H.W. designed the research and wrote the manuscript. S.K., D.D.M., P.D., and L.M performed experiments. S.S. performed mass spectrometric analysis. K.R. conceived and provided the peptide array.

## Supplemental Information

Table S1. HOIP-TAP, Analyzed by Mass Spectrometry, Related to Figure 1A HOIP-deficient K562 cells were reconstituted with TAP-tagged HOIP-WT, enzymatically inactive HOIP-C885S, or vector control. HOIP-containing complexes were purified and analyzed by mass spectrometry.

Table S2. TNFR1-SC Purification, Analyzed by Mass Spectrometry, Related to Figure 1B TNFR1-SC purification from A549 cells using TAP-TNF [500ng/ml]. Control cells were left untreated. Protein complexes were analyzed using mass spectrometry.
